# Evolution and long‑term respiratory sequelae after severe COVID-19 pneumonia: nitric oxide diffusion measurement value

**DOI:** 10.1186/s12931-023-02344-2

**Published:** 2023-02-13

**Authors:** Marta Núñez-Fernández, Cristina Ramos-Hernández, Francisco García-Río, Alexandre Pérez-González, Amara Tilve-Gómez, Paula Rodríguez-Fernández, Andrés Nodar-Germiñas, Alberto Fernández-García, Alberto Ruano-Raviña, Alberto Fernández-Villar

**Affiliations:** 1 Service of Pneumology, University Hospital Complex of Vigo, Vigo, Spain; 2 NeumoVigo I+i, Galicia Sur Health Research Institute (IISGS), Vigo, Spain; 3 Service of Pneumology La Paz-IdiPAZ University Hospital, Madrid, Spain; 4 CIBER Respiratory Diseases (CIBERES), Madrid, Spain; 5 Department of Medicine, University Autónoma de Madrid, Madrid, Spain; 6 Group of Virology and Pathogenesis, Galicia Sur Health Research Institute (IIS), Vigo, Spain; 7 Infectious Diseases Unit, Department of Internal Medicine, Galicia Sur Health Research Institute (IISGS), University Hospital Complex of Vigo, Vigo, Spain; 8 Service of Radiodiagnosis, University Hospital Complex of Vigo, Vigo, Spain; 9 Diagnostic Imaging Research Group, Galicia Sur Health Research Institute (IISGS), Vigo, Spain; 10 Service of Radiodiagnosis, Ribera-POVISA Hospital, Vigo, Spain; 11 Department of Preventive Medicine and Public Health, University of Santiago de Compostela, Santiago de Compostela, Spain; 12 Consortium for Biomedical Research in Epidemiology and Public Health (CIBERESP), Madrid, Spain

**Keywords:** COVID-19, Pneumonia, Sequelae, Respiratory function tests, Diffusion capacity, DLCO, DLNO, Long-term sequelae

## Abstract

**Introduction:**

There are no published studies assessing the evolution of combined determination of the lung diffusing capacity for both nitric oxide and carbon monoxide (DL_NO_ and DL_CO_) 12 months after the discharge of patients with COVID-19 pneumonia.

**Methods:**

Prospective cohort study which included patients who were assessed both 3 and 12 months after an episode of SARS-CoV-2 pneumonia. Their clinical status, health condition, lung function testings (LFTs) results (spirometry, DL_NO_-DL_CO_ analysis, and six-minute walk test), and chest X-ray/computed tomography scan images were compared.

**Results:**

194 patients, age 62 years (P_25–75_, 51.5–71), 59% men, completed the study. 17% required admission to the intensive care unit. An improvement in the patients’ exercise tolerance, the extent of the areas of ground-glass opacity, and the LFTs between 3 and 12 months following their hospital discharge were found, but without a decrease in their degree of dyspnea or their self-perceived health condition. DL_NO_ was the most significantly altered parameter at 12 months (19.3%). The improvement in DL_NO_-DL_CO_ mainly occurred at the expense of the recovery of alveolar units and their vascular component, with the membrane factor only improving in patients with more severe infections.

**Conclusions:**

The combined measurement of DL_NO_-DL_CO_ is the most sensitive LFT for the detection of the long-term sequelae of COVID-19 pneumonia and it explain better their pathophysiology.

## Introduction

The development of respiratory sequelae in patients surviving pneumonic processes caused by the SARS-CoV-2 has been a matter of particular concern and interest since the beginning of the pandemic [1, 2]. Numerous studies analyzing the short- and mid-term (first 6 months) persistence of clinical, radiological, and functional alterations have been published to date, with remarkable differences in their results (presence in 20–80% of the patients under study) depending on the patient populations included, the pneumonia severity, the type of supportive treatment administered and the methodology used [3–6]. The main clinical sequelae are persistent dyspnea, limitations on physical effort and general health status alterations. At a radiological level, the most frequently reported sequelae are the presence of areas of ground-glass opacity and either reticular lesions or parenchymal bands. With respect to the lung function, the most common findings are alterations in the diffusing capacity for carbon monoxide (DL_CO_) and a decrease in total lung capacity [3–11]. However, to date there is only scare and heterogeneous evidence available on the longer-term sequelae (≥ 1 year from hospitalization) [3–18]. Concerning the DL_CO_ decrease, there is considerable debate, owing to the reference values used, the interpretation of the carbon monoxide transfer coefficient (K_CO_) and the alveolar volume (VA), or the use of the reference percentage (%) instead of the lower limit of normality (LLN) [7, 8, 19, 20]. Another limitation of the DL_CO_ determination is that it does not allow for differentiating whether the gas exchange alterations are mostly caused by the involvement of the membrane component or that of the vascular component while this information could be obtained through the combined determination of the diffusing capacity for both carbon dioxide and nitric oxide (DL_CO_ and DL_NO_) [7, 8, 10, 12, 19–22]. In the only two studies published on this matter, the authors concluded that the combined determination of DL_NO_ and DL_CO_ is more sensitive than that of the DL_CO_ alone for detecting functional sequelae and is more strongly correlated with the patients’ health condition and exertional capacity following an episode of COVID-19 pneumonia. However, this assessment was performed at a single moment following the episode of pneumonia in both studies [12, 21], with no data being available thus far on the evolution of the alterations in the different gas exchange components (membrane and vascular) over time.

Considering the above, the aim of this study is to compare the clinical, radiological and functional sequalae at 3 and 12 months, using the combined determination of DL_NO_ and DL_CO_ of a large cohort of patients who were hospitalized for SARS-CoV-2 pneumonia. On the other hand, we also intend to analyse if this evaluation provides any additional information to improve the knowledge of these sequelae and their long-term evolution.

## Methods

This is a longitudinal follow-up study of a cohort of surviving patients with severe COVID pneumonia, in which two cross-sectional analyses were performed, at 3 and 12 months after hospital discharge. Part of the methods applied in this study have already been previously published [12].

As described previously [12], inclusion criteria were an age between 18 and 90 years, and an hospital discharge for COVID-19 pneumonia with evidence of alveolar condensation on a chest X-ray and a positive PCR result for SARS-CoV-2 in the nasopharyngeal swab or bronchoalveolar lavage. Were excluded all patients who received institutional care (eg, nursing homes or seriously disabled), as well as people who refused to or they were unable to sign the informed consent document. The study was approved by the Clinical Research Ethics Committee of Galicia (registration number 245/2020) and all participants signed an informed consent form.

All participants underwent an overall clinical assessment, determinations of LDH and D-dimer levels, radiology studies and lung function tests (LFTs) both 3 (± 1 week) and 12 (± 2 weeks) months following their hospital discharge.

The sociodemographic variables, smoking history, most significant comorbidities, overall health status prior to the admission based on the Eastern Cooperative Oncology Group (ECOG) scale, arterial oxygen saturation at admission, peak lactate dehydrogenase (LDH) and D-dimer levels during admission, Pneumonia Severity Index (PSI) prognostic scale score, unilateral or bilateral lung involvement, need for admission to the intensive care unit (ICU), and the length of hospitalization were recorded.

The degree of dyspnea was determined according to the modified Medical Research Council (mMRC) scale. The patients’ health status was assessed using the Spanish version of the Nottingham Health Profile (NHP) [23].

The LFTs were carried out using a MasterScreen PFT system (Viasys, CareFusion, Würzbourg, Germany) equipped with the SentrySuite™ software, including a forced spirometry test conducted following the joint recommendations of the American Thoracic Society and European Respiratory Society (ATS/ERS) [24] and using the Global Lung Function Initiative (GLI) equations [25] as reference values. Using this same equipment, the DL_NO_ and DL_CO_ were measured simultaneously during a single breath maneuver according to the ERS recommendations [26]. Both the membrane component diffusing capacity for carbon monoxide (DM_CO_) and the total capillary blood volume exposed to alveolar gas (Vc) were calculated using the model proposed by Guénard et al. [27].

A duplicate six-minute walk test (6MWT) was performed following the ATS recommendations [28].

All patients underwent a chest X-ray with two projections whose findings were reported by consensus by two expert radiologists. These findings were categorized into complete resolution (normal study or with findings similar to those observed during the previous admission) or incomplete resolution.

Patients with a dyspnea mMRC grade ≥ 2, radiographic abnormalities, and/or a DL_CO_ < LLN underwent a chest computed tomography (CT) scan within the following 2 weeks. The CT scan images were examined by two expert radiologists who were blinded to the patients’ clinico-functional status. The presence of areas of ground-glass opacity, reticular lesions, bronchiectasis, and a honeycomb pattern in these images were recorded. The extent of the lesions was calculated from the mean value of two visual assessments of the involvement of all five lung lobes, we used a score previously described [14, 29].

### Statistical analysis

Normality of quantitative variables was evaluated using the Shapiro–Wilk test and their values were expressed as a median and interquartile range. Qualitative variables were expressed as a number and percentage. Numerical variables collected at 3 and 12 months were compared using the Wilcoxon signed-rank test, whereas qualitative variables were compared using McNemar’s test. Finally, the comparison of variables according to ICU admission was performed using the Mann–Whitney U test. Statistical package SPSS for Windows, version 25 (IBM Corp, Armonk, NY, USA) was used for all analyses.

## Results

Of the 210 evaluated subjects, a total of 194 patients who were able to undergo a valid and reproducible clinical evaluation and spirometric tests 3 and 12 months following their hospital discharge were included in the study. Their main characteristics are summarized in Table 1.

**Table 1:** Sociodemographic, clinical, and general characteristics of the pneumonic process caused by COVID-19 in the patients included in the study

Variables	Total
Demografics and clinics before admission	N = 194
Male sex, N (%),	114 (55.8)
Age, years	62 (51.5–71)
Body mass index (Kg/m^2^)	29 (26–32)
Previous and current smoker, N (%)	78 (40.2)
History of chronic cardiopathy, N (%)	36 (18.6)
History of diabetes, N (%)	21 (10.8)
History of hypertension, N (%)	71 (36.6)
History of COPD, N (%)	13 (6.7)
History of chronic kidney failure, N (%)	6 (3.1)
ECOG score	1 (1–2)
ECOG score ≥ 2, N (%)	51 (26.2)
*In relation to the pneumonic process*	*N = 194*
Bilateral radiographic involvement, N (%)	149 (76.8)
Oxygen saturation at hospital admission	92 (89–97)
Pneumonia Severity Index	61 (50–71)
Pneumonia Severity Index ≥ 3, N (%)	62 (31.9)
ICU admission, N (%)	33 (17)
Invasive mechanical ventilation, N (%)	26 (13.4)
Maximum level of lactate deshydrogenase (U/L)	294 (230–386.5)
Maximum level of D-dimer (ng/mL)	1040 (486.5–2544.5)
Length of stay (days)	7 (4–13.2)

Median (25th and 75th percentile)

*COPD* chronic obstructive pulmonary disease, *ECOG* Eastern Cooperative Oncology Group, *ICU* intensive care unit

Table 2 shows a comparison of the clinical, functional, and radiological determinations performed, including the number of subjects who underwent each of these tests at 3 and 12 months. No significant differences were observed in their grade of dyspnea or in any of the NHP domains. However, a decrease was observed in the number of patients presenting with chest X-ray alterations, in addition to a reduction in the extent of the lesions resulting from a decrease in the size of the areas of ground-glass opacity, albeit not in that of the reticular lesions or the bronchiectasis, in the 70 patients who underwent a thoracic CT scan at 3 and 12 months. A significant improvement was also observed in the patients’ exercise tolerance, with an increase in the distance covered during the 6MWT, which exceeded the minimum clinically relevant difference of 30 m in 106 (61.6%) patients.

**Table 2:** Comparison in clinical, functional, laboratory parameters and radiological situation at 3 and 12 months after hospital discharge

Variables	At 3 months	At 12 months	P
*Dyspnea according to mMRC*	*N = 194*	*N = 194*	
Dyspnea ≥ 1, N (%)	89 (45.9)	77 (39.7)	0.38
Dyspnea ≥ 2, N (%)	20 (10.3)	21 (10.8)	0.70
Dyspnea	0 (0–1)	0 (0–1)	0.84
*Health status according to Nottingham Health Profile*	*N = 189*	*N = 189*	
Energy	0 (0–33)	0 (0–33)	0.53
Pain	0 (0–40.6)	0 (0–37.5)	0.72
Physical mobility	12.5 (0–37)	12.5 (0–37.5)	0.52
Emotional reactions	11.1 (0–33.3)	11.1 (0–33.3)	0.61
Sleep	20 (0–60)	20 (0–60)	0.72
Social isolation	0 (0–0)	0 (0–0)	0.89
Number of limited areas	3 (2–4)	3 (1–5)	0.64
*Spirometry*	*N = 194*	*N = 194*	
FVC, % of predicted	103 (92–115)	107 (98–117)	0.0001
FVC < LLN, N (%)	10 (5.2)	3 (1.6)	0.03
FEV_1_, % of predicted	103 (92–115)	105 (93–115)	0.32
FEV_1_ < LLN, N (%)	15 (7.7)	7 (3.6)	0.02
*Gas difusión*	*N = 189*	*N = 187*	
DL_NO_, % of predicted	79 (68.5–87.5)	83 (73–93)	0.0001
DL_NO_ < LLN, N (%)	55 (29.1)	36 (19.3)	0.02
DL_CO_, % of predicted	86 (74–97)	94 (83–109)	0.0001
DL_CO_ < LLN, N (%)	32 (16.9)	18 (9.6)	0.02
VA, % of predicted	87 (78.5–94)	97 (89–110)	0.0001
K_NO_, % of predicted	90 (83–99)	86 (80–93)	0.0001
K_NO_ < LLN, N (%)	18 (9.5)	21 (11.2)	0.39
K_CO_, % of predicted	96 (84–105)	93 (83–102)	0.0001
K_CO_ < LLN, N (%)	10 (5.3)	10 (5.3)	0.99
DM_CO_, % of predicted	69 (57–79.5)	71 (60–81)	0.001
DM_CO_/VA, % of predicted	83 (74–93.7)	78 (69–86)	0.0001
Vc, % of predicted	85 (74–98)	97 (84–110)	0.0001
Vc/VA, % of predicted	101 (88–112.5)	100 (89–113)	0.40
DL_NO_/DL_CO_	4.5 (4.3–4.6)	4.3 (4.2–4.5)	0.0001
DM_CO_/Vc	1.4 (1.2–1.7)	1.3 (1.1–1.5)	0.0001
*Exercise capacity (6MWT)*	*N = 181*	*N = 179*	
6-min walking distance, m	456 (391–521)	510 (465–558)	0.0001
Initial oxygen saturation, %	98 (97–99)	98 (97–98)	0.08
Final oxygen saturation, %	97 (95–98)	97 (96–97)	0.23
Initial Borg scale dyspnea (1–10)	0 (0–0)	0 (0–0)	0.64
Final Borg scale dyspnea (1–10)	2 (0–4)	1 (0–5)	0.41
*Laboratory parameters*	*N = 192*	*N = 192*	
D-dimer, ng/mL	339 (225–565-5)	328 (315–515)	0.09
Lactate deshydrogenase, U/L	195 (173–217)	191 (163–308)	0.06
*Chest X-ray*	*N = 194*	*N = 194*	
Persistence of any lung injuries, N (%)	54 (27.8)	11 (5.7)	0.0001
*Chest CT*	*N = 87*	*N = 70*	
Score extension affectation	6 (0–11)	5 (3–8)	0.0001
Ground glass opacities > 25%, N (%)	27 (31)	13 (18.6)	0.0001
Presence of reticulation, N (%)	24 (27.6)	21 (30)	0.38
Presence of bronchiectasis, N (%)	18 (20.7)	20 (28.6)	0.80

Median (25th and 75th percentile). Wilcoxon test

*FVC* forced vital capacity, *LLN* lower limits of normal, *FEV1* forced expiratory volume in first one second, *DL*_*NO*_ diffusion capacity of nitric oxide, *DL*_*CO*_ diffusion capacity of carbon monoxide, *VA* alveolar lung volume, *K*_*NO*_ rate of uptake of nitric oxide from alveolar gas, *K*_*CO*_ rate of uptake of carbon monoxide from alveolar gas, *DM*_*NO*_ membrane conductance of nitric oxide, *VC* pulmonary capillary blood volume, *6MWT* six-minute walk test, *CT* computerized tomography

Slight improvements were also detected in the forced vital capacity (FVC) and the forced expiratory volume within the first second (FEV_1_), as well as a lower proportion of patients with a decreased FVC (< LLN), which persisted in only 1.5% of the patients one year after their recovery from the episode of pneumonia.

A comparison of the results of the diffusion study performed at 3 and 12 months revealed an improvement in the DL_NO_, but especially in the DL_CO_ (thus causing a significant reduction in the DL_NO_/DL_CO_ ratio), with the DL_NO_ being the most frequently altered parameter at 12 months (19.3% of patients). The 18 patients who had a DL_CO_ < LLN at 12 months also had a DL_NO_ < LLN. An increase of over 10% in the DL_NO_ and the DL_CO_ was observed in 51 (27%) and 70 (37.4%) patients, respectively.

**Fig. 1:**
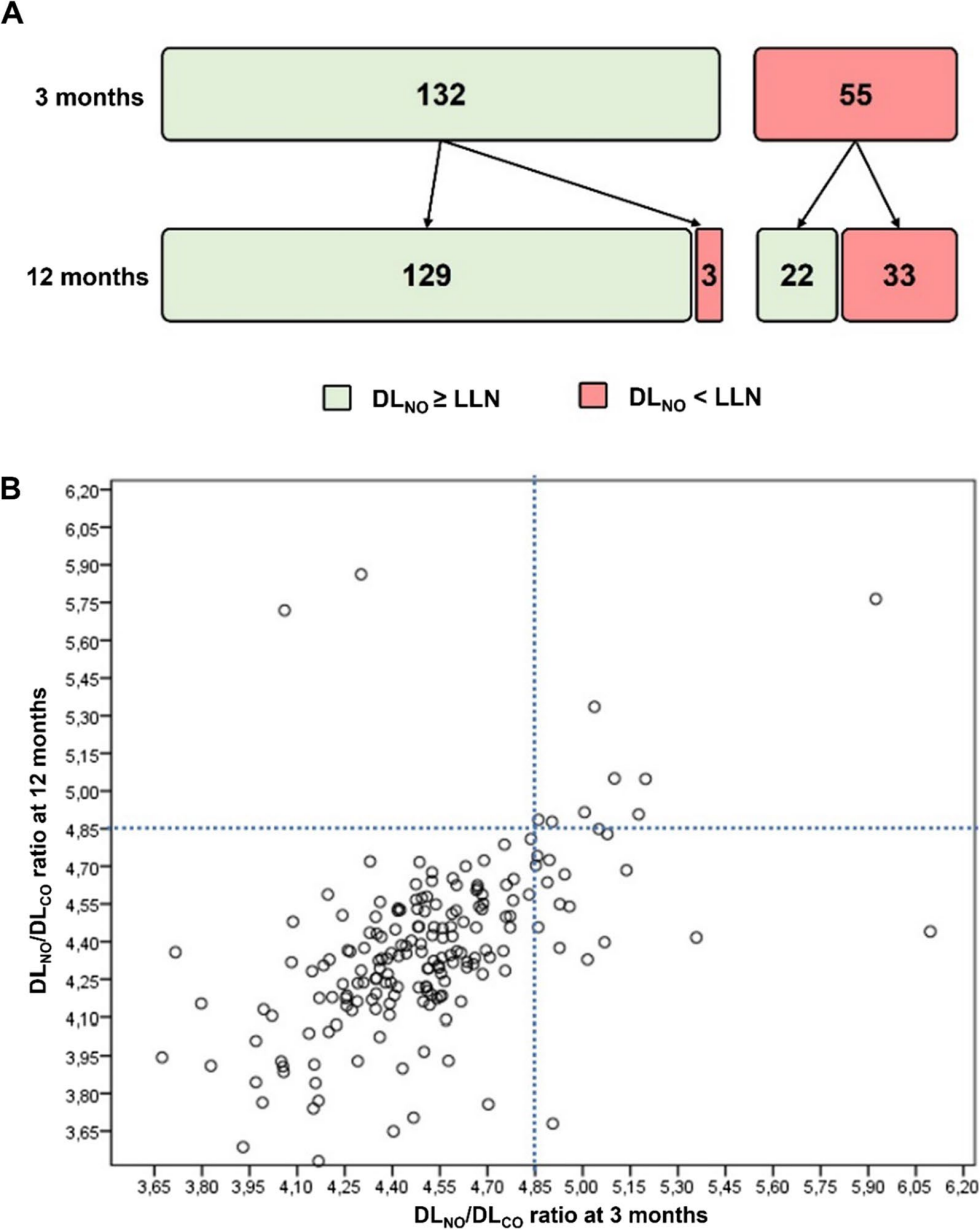
Number of patients with decreased DL_NO_ at 12 months based on their status at 3 months (**A**) and the concordance of the DL_NO_/DL_NO_ ratio between 3 and 12 months (**B**). *DL*_*NO*_ diffusion capacity of nitric oxide, *DL*_*CO*_ diffusion capacity of carbon monoxide, *LLN* lower limits of normal

Figure 1 shows the number of patients with decreased DLNO at 12 months based on their status at 3 months (Fig. 1A) and the concordance of the DLNO/DLNO ratio between 3 and 12 months (Fig. 1B), in this case, using the cut-off point of 4.85 proposed by Zavorsky et al. [26]

On the other hand, a remarkable increase in the VA was detected in the 187 patients who underwent diffusion studies during both follow-up periods, with the mean VA raising from 4.9 L at 3 months to 5.4 L at 12 months. This increase was greater than that of the diffusion capacity, in such a way that the mean K_NO_ and K_CO_ values decreased slightly, but the number of patients with a low K_NO_ or K_CO_ remained practically unchanged. As for the diffusion components, their greatest increase occurred at the expense of the Vc (≈14% between 3 and 12 months) and was much more discrete for the DM_CO,_ whose increase was significantly lower that of the VA.

Table 3 reflects the differences between the lung function parameters at 3 and 12 months following the discharge as a function of the ICU admission. Patients admitted to the ICU exhibited a greater improvement in their DL_NO_, DL_CO_, VA, and six minutes walked distance (6MWD). No differences were found in the evolution of the DL_NO_/DL_CO_ ratio between both subgroups, and although the increase in the DM_CO_ was greater in those patients who had to be admitted to the ICU, neither the change adjusted for the VA nor the Vc variation differed between the two patient subgroups.

**Table 3:** Differences in the LFT between 3 and 12 months depending on ICU admission

Variables	All patients	Non ICU	ICU	P
*Spirometry*	*N = 194*	*N = 161*	*N = 33*	
Change in FVC, % of predicted	7 (5–13)	7 (0–12)	8 (2.5–15.5)	0.14
Change in FEV_1_, % of predicted	5 (− 1–11)	5 (− 1–11)	7 (0.5–13.5)	0.16
*Gas difusión*	*N = 187*	*N = 156*	*N = 31*	
Change in DL_NO_, % of predicted	5 (− 1–11)	4 (− 1–11)	7 (3–11)	0.02
Change in DL_CO_, % of predicted	8 (1–14)	7 (0–12.2)	11 (6.5–17)	0.01
Change in VA, % of predicted	10 (3–15)	10 (6–14)	13 (8–16.5)	0.03
Change in K_NO_, % of predicted	− 5 (− 8–1)	− 5 (− 8–2)	− 4 (− 6–1)	0.13
Change in K_CO_, % of predicted	− 2 (− 6–2)	− 2 (− 6–2)	0 (− 5–4)	0.15
Change in DM_CO_, % of predicted	1 (− 3–10)	1 (− 4–9)	6 (0–10.5)	0.04
Change in DM_CO_/VA, % of predicted	− 6 (− 12–0)	− 6 (− 12–0)	− 5 (− 9–2)	0.16
Change in Vc, % of predicted	9 (2–16)	8.5 (2–15)	9 (4–18)	0.40
Change in Vc/VA, % of predicted	− 1 (− 7–7)	− 1 (− 7–6.5)	− 1 (− 9–7)	0.98
Change in DL_NO_/DL_CO_	− 0.13 (− 0.20–0.00)	− 0.13 (− 0.31–0.01)	− 0.14 (− 0.27–0.01)	0.90
Change in DM_CO_/Vc	− 0.08 (− 0.25–0.07)	− 0.09 (− 0.28–0.07)	− 0.25 (− 0.22–0.09)	0.42
*Exercise capacity (6MWT))*	*N = 172*	*N = 143*	*N = 29*	
Change in walking distance, m	49.5 (7.2–87.7)	44 (3–80)	77 (33–121)	0.03
Difference initial oxygen saturation, %	− 1 (− 1–0)	− 1 (− 1–0)	− 1 (− 1–0)	0.90
Difference final oxygen saturation, %	0 (− 1.7–1)	− 1 (− 2–1)	0 (− 1–1)	0.30

Median (25th and 75th percentile). Mann–Whitney test

*LFT* lung function testing, *ICU* intensive care unit, *FVC* forced vital capacity, *FEV1* forced expiratory volume in first one second, *DL*_*NO*_ diffusion capacity of nitric oxide, *DL*_*CO*_ diffusion capacity of carbon monoxide, *VA* alveolar lung volume, *K*_*NO*_ rate of uptake of nitric oxide from alveolar gas, *K*_*CO*_ rate of uptake of carbon monoxide from alveolar gas, *DM*_*CO*_ membrane conductance of carbon monoxide, *Vc* pulmonary capillary blood volume, *6MWT* six-minute walk test

Finally, a persistently decreased DL_NO_ 12 months after the patients’ admission for severe pneumonia was associated with a higher percentage of patients with a dyspnea grade ≥ 2, less 6MWD, a lower oxygen saturation both at baseline and after the six minutes of walking, and higher D-dimer levels (Table 4). However, no differences were observed in the health status dimensions and the thoracic CT scan images only revealed remarkable differences in the presence of reticulation, although this test was only performed in 44% and 61% of the patients with normal or reduced DL_NO_, respectively.

**Table 4:** Degree of dyspnea, state of health, exercise capacity, analytical determinations and radiological involvement at 12 months depending on the presence or absence of DL_NO_ < LLN

Variables	DL_NO_ ≥ LLN	DL_NO_ < LLN	P
*Dyspnea according to mMRC*	*N = 151*	*N = 36*	
Dyspnea ≥ 1, N (%)	58 (38.4)	14 (41.7)	0.60
Dyspnea ≥ 2, N (%)	14 (9.3)	7 (20.4)	0.04
Dyspnea	0 (0–1)	0 (0–1)	0.42
*Health status according to Nottingham Health Profile*	*N = 150*	*N = 33*	
Energy	0 (0–33.3)	0 (0–33.3)	0.15
Pain	12.5 (0–37.5)	12.5 (0–25)	0.33
Physical mobility	12 (0–37.5)	25 (0–37.5)	0.72
Emotional reactions	11.1 (0–33.3)	11.1 (0–33.3)	0.70
Sleep	20 (0–60)	20 (0–60)	0.52
Social isolation	0 (0–0)	0 (0–1)	0.46
Number of limited areas	3 (1–5)	3 (1–5)	0.95
*Exercise capacity (6MWT)*	*N = 142*	*N = 34*	
Distance, m	519.5 (472.5–562.2)	487.5 (359.7–547.7)	0.02
Initial oxygen saturation, %	98 (97–98)	97 (96–98)	0.004
Final oxygen saturation, %	97 (96–98)	96 (94–97)	0.001
Initial Borg scale dyspnea (1–10)	0 (0–1)	0 (0–1)	0.16
Final Borg scale dyspnea (1–10)	2 (0–5)	2 (0–4)	0.40
*Laboratory parameters*	*N = 151*	*N = 35*	
D-dimer, ng/mL	312 (215–458)	395 (253–738)	0.01
Lactate deshydrogenase, U/L	192 (173–211)	184 (173–203)	0.10
*Chest X-ray*	*N = 151*	*N = 37*	
Persistence of any lung injuries, N (%)	9 (6)	2 (5.6)	0.9
*Chest CT*	*N = 44*	*N = 22*	
Score extension affectation	5.5 (2–8.7)	4 (3–8.2)	0.84
Ground glass opacities > 25%, N (%)	8 (18.2)	4 (18.2)	0.90
Presence of reticulation, N (%)	10 (22.7)	10 (45.4)	0.05
Presence of bronchiectasis, N (%)	13 (29.5)	7 (31.8)	0.95

Median (25th and 75th percentile). Mann–Whitney test

*DL*_*NO*_ diffusion capacity of nitric oxide, *LLN* lower limits of normal, *mMRC* Modified Medical Research Council, *6MWT* six-minute walk test, *CT* computerized tomography

## Discussion

This is the first study analyzing the mid- and long-term clinical, radiological, and functional evolution using the combined assessment of DL_NO_ and DL_CO_ in patients hospitalized for SARS-CoV-2 pneumonia, providing additional and novel information about the potential sequelae of the pneumonic episode and their pathophysiologic mechanisms [10]. The main conclusion reached are that between 3 and 12 months, patients experience an improvement in their exercise capacity, but not in their perception of dyspnea nor in their health status. A decrease in the size of the radiological lesions that persisted at 3 months was also observed and, concerning the LFTs, the most remarkable findings were the increase in the parameters related to the vascular diffusion component and the recovery of alveolar units, with the most frequent persistent alterations being related to the membrane component, which exhibited greater improvements in patients who experienced more severe pneumonic episodes. Patients with DL_NO_ alterations at 12 months had higher dyspnea grades, a lower oxygen saturation at rest, lower exercise tolerance, and higher D-dimer levels.

Almost 40% of our patients reported some degree of dyspnea at 12 months, similar to other series [13, 14, 16]. Concerning the health status, our patients reported alterations mainly in the mobility, emotional, and sleep quality domains, which could be due to the high perception of dyspnea despite the increase in exercise capacity between 3 and 12 months, a finding that has also been described by other authors [15]. The relationship between persistent dyspnea and a high impact on the psychoemotional domains of different questionnaires has also been described in other studies on long-term sequelae [13–17].

Regarding the evolution of alterations in the thoracic CT scans at 3 months, we observed a decrease in the extent of the areas of ground-glass opacity, but not in other lesions, such as reticulation or bronchiectasis. 12 months after hospital discharge, approximately 34% and 30% of our patents continued to have some areas of ground-glass opacity and either reticular lesions or bronchiectasis, respectively, similarly to described by other authors [13, 15–18], which indicates that the mid-term radiological alterations tend to improve or remain unchanged, without signs of progression to fibrosis in the majority of patients.

In agreement with previous findings [14–18], the spirometric impact of COVID-19 was very low, and the most important alterations affected gas exchange, confirming the results reported by Barisione and Brusasco [21], who evaluated the combined analysis of DL_NO_ and DL_CO_ in a series of 94 patients without other comorbidities who underwent a single assessment 10–266 days after recovering from COVID-19. As in our patients, DL_NO_ alterations were more frequent than DL_CO_ ones regardless of the time elapsed since the infectious episode, and the DL_CO_ was proportionally less decreased among those patients who had recovered from the disease over 3 months earlier. This suggests that the decreased diffusion might primarily be explained by a DM_CO_ reduction secondary to the damage and loss of alveolar units, while the Vc improved more proportionally over time, even in patients with little persistent involvement in the follow-up thoracic CT scans [21]. Our study provides additional information to better determine the longitudinal evolution of post-COVID-19 sequelae. We found that the DL_NO_/DL_CO_ ratio (a reflection of the DM_CO_/Vc ratio) decreased significantly between 3 and 12 months after hospital discharge, and that the DM_CO_ was the most severely affected variable exhibiting the least improvement. This finding, together with the striking increase in both the VA and Vc, seems to confirm that the loss of functional alveolar units secondary to the pneumonia partially recovers within the following months, mainly at the expense of the perfusion component, with the reduction in the alveolar surface area being greater than the microvascular damage, which consequently results in a greater impact on the DL_NO_ than the DL_CO_. This alteration could obviously be due to localized alveolar destruction with a certain degree of fibrosis, but also to circumstances that are more easily reversible and persistent within the months following the episode of pneumonia, such as infiltration, exudate, or edema [10, 30]. As an exception to this general behavior, in our study we found that the improvement in the membrane component was greater among those patients who had more severe pneumonic conditions (ICU admission), probably as a result of a reversal of the damage to the alveolocapillary barrier caused by the acute respiratory distress. In fact, because this difference disappeared when correcting the changes in the DM_CO_ for the VA, it could be exclusively attributable to the gradual reopening of collapsed alveolar units.

The higher percentage value of the K_CO_ compared with the DL_CO_, as well as its stable or even slightly decreasing trend, was also observed in other studies with a 6- and 12-month follow-up [14, 31], and might be explained by the complex relationship between the K_CO_ and VA, as well as by the fact that the DL_CO_ improvement might not affect a small percentage of patients who continue to have a persistently low K_CO_ [10, 32]. In the absence of inspiratory pressure measurements, the K_CO_ values suggest that it is unlikely that the decrease in the initial VA and its improvement are related, or at least significantly, to a recovery of alveolar expansion due to muscle weakness, as this would also affect the DL_NO_/DL_CO_ ratio in other ways.

The number of patients with a low DL_CO_ at 12 months observed in this study was significantly lower than that found in others (25–60%) [13, 17], albeit similar to that reported by Chen et al. [18]. Although some authors[7] argue that these differences could be explained by the different cut-off points (< LLN versus < 80% of the predicted value), reference values, populations, or pneumonia severity grades used in each study, it must be noted that we started from a number of cases with a lower DL_CO_ alteration at 3 months than most of the studies with which we compared our findings, although we believe that this variability requires more specific research.

In the long-term, the identification of a persistently reduced DL_NO_ allows for detecting patients with a greater degree of dyspnea and desaturation, both at rest and during exercise, in addition to those with radiological lesions less susceptible to improvement, such as reticular alterations. These results are superimposable to those exhibited by patients with a low DL_CO_ (data not shown), although they only represent half of the cases; therefore, the combined determination of DL_NO_ and DL_CO_ could better explain the persistence of severe dyspnea or limitations on physical effort than the determination of DL_CO_ itself, in addition to providing further information on patients with the aforementioned alterations and a DL_CO_ within the limits of normality [10, 21].

This study has some limitations. Firstly, it was carried out in a single hospital, which, despite potentially limiting its external validity, provides consistency, especially to the results of the LFTs performed at two different time points by the same professionals and using the same equipment. Secondly, certain tests that have also been shown to yield altered results in the long term in patients with COVID-19 pneumonia and that could complement the information provided by this research [13–18], such as the determination of pulmonary volumes, were not performed in this study, although these parameters were determined by the diffusion study itself in some study [13]. Finally, a thoracic CT scan was performed in only some cases, although the results obtained in the study are mostly in line with those published to date.

Notwithstanding the foregoing, it also has some strengths, as it presents one of the most important case studies of the few published to date in this respect, a low loss of cases in relation to the conduct of the functional tests, completed by over 90% of the patients, and the diffusion study was performed using a technique that, despite not being widespread, is standardized and provides information to better understand the sequelae of a new disease that has affected hundreds of millions of people worldwide [7, 10, 21].

In conclusion, between 3 and 12 months after an episode of SARS-CoV-2 pneumonia, patients exhibit a remarkable recovery in their exercise tolerance and gas exchange, both of which are mainly attributable to the recovery of functional alveolar units and the vascular diffusion component, although an improvement in the membrane component is also observed in more severe cases. However, functional improvement does not translate into an improvement in the patients’ clinical condition and/or perceived health status with respect to that observed at 3 months of discharge. Further studies and a longer-term follow-up are needed to confirm these findings and to better understand the respiratory sequelae and their clinical consequences in patients who developed pneumonia during a SARS-CoV-2 infection.

### Take-away message

DL_NO_–DL_CO_ is the most sensitive LFT for the detection of the long-term sequelae of severe COVID-19 pneumonia and provides information on the pathophysiology of its recovery.

## Data Availability

Database is available upon reasonable request to any author, editor, or reviewer wishing to use it.
